# Association between circadian activity rhythms and mood episode relapse in bipolar disorder: a 12-month prospective cohort study

**DOI:** 10.1038/s41398-021-01652-9

**Published:** 2021-10-13

**Authors:** Yuichi Esaki, Kenji Obayashi, Keigo Saeki, Kiyoshi Fujita, Nakao Iwata, Tsuyoshi Kitajima

**Affiliations:** 1 Department of Psychiatry, Okehazama Hospital, Aichi, Japan; 2 Department of Psychiatry, Fujita Health University School of Medicine, Aichi, Japan; 3 Department of Epidemiology, Nara Medical University School of Medicine, Nara, Japan; 4 The Neuroscience Research Center, Aichi, Japan

**Keywords:** Bipolar disorder, Human behaviour

## Abstract

A significant proportion of patients with bipolar disorder experience mood episode relapses. We examined whether circadian activity rhythms were associated with mood episode relapses in patients with bipolar disorder. This prospective cohort study included outpatients with bipolar disorder who participated in a study titled “Association between the Pathology of Bipolar Disorder and Light Exposure in Daily Life (APPLE) cohort study.” The participants’ physical activity was objectively assessed using a wrist-worn accelerometer over 7 consecutive days for the baseline assessment and then at the 12-month follow-up for mood episode relapses. The levels and timing of the circadian activity rhythms were estimated using a cosinor analysis and a nonparametric circadian rhythm analysis. Of the 189 participants, 88 (46%) experienced mood episodes during follow-up. The Cox proportional hazards model adjusting for potential confounders showed that a robust circadian activity rhythm, including midline-estimating statistic of rhythm (MESOR) and amplitude by cosinor analysis and 10 consecutive hours with the highest amplitude values (M10) by the nonparametric circadian rhythm analysis, was significantly associated with a decrease in mood episode relapses (per counts/min, hazard ratio [95% confidence interval]: MESOR, 0.993 [0.988–0.997]; amplitude, 0.994 [0.988–0.999]; and M10, 0.996 [0.993–0.999]). A later timing of the circadian activity rhythm (M10 onset time) was significantly associated with an increase in the depressive episode relapses (per hour; 1.109 [1.001–1.215]). We observed significant associations between circadian activity rhythms and mood episode relapses in bipolar disorder.

## Introduction

Bipolar disorder is a severe, chronic, recurrent mental illness characterized by depressive, manic, and hypomanic mood episodes. Over one-third of patients with bipolar disorder relapse within 1 year despite maintenance therapy [1], with >90% experiencing at least one additional mood episode during their lifetime [2]. Mood episodes are associated with a socioeconomic burden, a caregiver burden, reduced quality of life, and increased suicidal tendencies [3, 4]. The factors associated with mood episode relapses in bipolar disorder therefore need to be identified.

Circadian activity rhythm disruption is a core feature in bipolar disorder, and there are studies reporting abnormal circadian activity rhythms, including lower amplitude, phase advance or delay, and less stability, in patients with bipolar disorder on comparison with healthy controls [5–7]. Additionally, circadian activity rhythm is closely associated with mood symptoms. A systematic review of patients with bipolar disorder suggested that exercise was associated with positive effects on depressive symptoms, quality of life, and functioning [8]. A prospective cohort study of 80 patients with bipolar disorder reported that reduced physical activity measured using a questionnaire was associated with mood episode relapse and hospitalizations for psychiatric disorders [9]. The evidence indicates that physical activity levels are associated with mood symptoms in bipolar disorder. Furthermore, the timing of physical activity is also associated with mood symptoms. A previous study reported that the severity of depressive symptoms was associated with the evening chronotype [10]. Another study demonstrated that comorbid circadian rhythm sleep–wake disorders, mainly delayed sleep–wake phase disorder, could be a significant predictor of relapse in bipolar disorder [11]. Based on these factors, it is likely that the level and timing of physical activity is associated with mood episode relapses in bipolar disorder. To our knowledge, however, no previous study has investigated whether objectively measured circadian activity rhythms, including level, timing, fragmentation, and stability, are associated with subsequent mood episodes in patients with bipolar disorder.

In this prospective cohort study, we examined the effect of circadian activity rhythms objectively measured using a wrist-worn accelerometer on mood episode relapses in bipolar disorder. We hypothesized that a lower level and later timing of circadian activity rhythms are associated with an increase in subsequent mood episodes.

## Materials and methods

### Participants and study design

The present study employed data from the “Association between the Pathology of Bipolar Disorder and Light Exposure in Daily Life (APPLE) cohort study.” Details on the APPLE cohort study have been provided elsewhere [12]. Briefly, the APPLE cohort study is a prospective, naturalistic observational study of outpatients with bipolar disorder conducted by two hospitals and two clinics in Japan since August 2017. The inclusion criteria were participants aged 18–75 years with bipolar disorder (I or II, according to the Diagnostic and Statistical Manual of Mental Disorders, Fifth edition [DSM-5]) diagnosed by an experienced psychiatrist. The present study excluded patients employed as night-shift workers, those with a serious risk of suicide as judged by a clinician, and those with acute mood episodes, including manic, mixed, and depressive episodes. During the baseline assessment at the clinic, the participants’ demographic and clinical characteristics were recorded, and they were asked to perform the following actions at home for 7 consecutive days: (1) wear an accelerometer (Actiwatch Spectrum Plus, Respironics, Pittsburgh, PA, USA) on the wrist of their nondominant arm for 24 h/day; and (2) record their bedtimes and rising times in a sleep diary. After the baseline assessment, the participants were followed up for 12 months for mood episode relapses. During the follow-up, each participant’s psychiatrist chose the medication and psychotherapy.

The authors assert that all procedures contributing to this work comply with the ethical standards of the relevant national and institutional committees on human experimentation and with the Helsinki Declaration of 1975, as revised in 2008. All procedures involving human patients were approved by the Ethics Committee of Okehazama Hospital (identifier: H29-011). Written informed consent was obtained from all patients before participating in the study. The study is registered at the University hospital Medical Information Network Clinical Trials Registry (UMIN-CTR identifier: UMIN000028239).

### Circadian activity rhythm assessment

Circadian activity rhythms were analyzed using the accelerometer at 1-min intervals 24 h/day throughout the study period. The detection algorithm in the accelerometer software (Actiware, version 6.0.9, Respironics) automatically excluded any periods during which the accelerometer was not worn; if any of these periods exceeded 10% of the 24 h period, the data for that day were excluded from the analysis. The percentage of such days was 12.4%. The main reasons due to which the accelerometer was not worn were discomfort in wearing it and forgetting to wear it after bathing.

### Mood episode relapses

Each participant’s psychiatrist assessed the participant’s mood episodes at each visit for 12 months from the baseline assessment and entered the data on the mood episodes into the medical record. The visit interval during the follow-up was chosen by each participant’s psychiatrist. At 12 months from the baseline assessment, one of the study authors (Y.E.) asked the participants’ psychiatrists to identify the participants’ mood episodes during the past 12 months based on their medical records. The mood episodes were diagnosed as depressive, manic, hypomanic, or mixed episodes according to the DSM-5 criteria. The study defined the time from the baseline assessment to the onset of the first mood episode as the time to a mood episode relapse; defined the time from the baseline assessment to the onset of the first depressive episode as the time to a depressive episode relapse; and defined the time from baseline assessment to the onset of the first manic, hypomanic, or mixed episode as the time to a manic/hypomanic/mixed episode relapse. The time to mood episode relapses was counted in months.

### Covariates

We defined the age at the first visit to a psychiatric clinic or hospital as the age at the onset of bipolar disorder. Each participant’s baseline depressive or manic status was assessed using the Montgomery–Åsberg Depression Rating Scale (MADRS) and the Young Mania Rating Scale (YMRS) [13, 14]. The International Society for Bipolar Disorders Task Force recommends a MADRS or YMRS score of <8 points to indicate symptomatic remission of bipolar depression or mania, respectively [15]. Our study therefore defined a MADRS or YMRS score ≥8 points as indicative of residual mood symptoms. Information on multiple mood episodes within 1 year before the baseline assessment were obtained from the participants’ psychiatrists. Sleep and daytime illuminance were objectively evaluated using accelerometer. Sleep periods were defined by a default threshold of <40 activity counts/min, which has been shown to be as accurate as polysomnography in measuring sleep in patients with bipolar disorder [16]. Total sleep time was defined as the total time spent asleep between bedtime and rising time, excluding wake after sleep onset. Sleep efficiency was defined as the percentage of total sleep time divided by the time between bedtime and rising time. Daytime illuminance was defined as the average daytime illuminance from rising time to bedtime.

### Statistical analysis

The statistical analyses were performed using R version 4.0.3 (R Core Team) and SPSS version 25.0 for Windows (IBM Corp., Armonk, NY, USA). The continuous variables are presented as medians and interquartile ranges (IQRs), and the categorical variables are presented as numbers and percentages. For the analysis, we employed the mean values for the sleep and light exposure data from the 7 consecutive measurement days. We compared the median values between the dichotomous groups using Mann–Whitney *U* test. We employed chi-squared test to compare the categorical data and considered a two-sided *P* < 0.05 to have statistical significance.

We performed the cosinor analysis using the R package “cosinor” and “cosinor2” (cosinor: http://github.com/sachsmc/cosinor; cosinor2: https://github.com/amutak/cosinor2). This analysis, the fitting of cosine curves to data using least-squares regression, has proven useful in characterizing the features of biological processes that exhibit an endogenous circadian component [17]. We first assigned the individual circadian activity rhythm periods by extracting a peak between *T* = 23 h and *T* = 25 h from the periodogram, given that one-third of patients with bipolar disorder have been reported to have the comorbidity of circadian rhythm sleep–wake disorder [18]. Using a single cosinor analysis, we then estimated the following three parameters: (1) the midline-estimating statistic of rhythm (MESOR), which is the mean activity level; (2) the amplitude, which is the height of the peak with respect to the mean level; and (3) the acrophase, which is the timing of the activity peak. The acrophase was corrected using the acrophase correction. This cosinor analysis method has been employed in a previous study of patients with bipolar disorder [19].

We performed the nonparametric circadian rhythm analysis using the R package “nparACT” [20]. This analysis complements the cosinor analysis by addressing rhythm fragmentation, stability, and mean activity levels and timing [21, 22]. We used the following seven parameters in this study: (1) interdaily stability; (2) intradaily variability; (3) least active continuous 5 h period (L5); (4) L5 onset; (5) most active continuous 10 h period (M10); (6) M10 onset; and (7) relative amplitude. The interdaily stability ranges from 0 to 1, with higher values indicating higher invariability of the interdaily 24 h rhythm. The intradaily variability ranges from 0 to 2, with higher values indicating higher fragmentation of the circadian rhythm. The relative amplitude is the difference between M10 and L5 divided by the sum of activity during these 15 h and ranges from 0 to 1, with higher values indicating a clearer distinction between activity levels during the most and least active periods of the day.

We estimated the time to mood episode relapse associated with circadian activity rhythm parameters using the Kaplan–Meier survival curves and the Cox proportional hazards model. In the Kaplan–Meier survival curves, the circadian activity rhythm parameters were analyzed using dummy variables, in which participants were divided into two groups (category based on the median value) according to each circadian activity rhythm parameter. In the Cox proportional hazards model, we analyzed the L5 parameter using natural log-transformed continuous variables given that it was not normally distributed. In multivariable analysis, we calculated the hazard ratio by simultaneously adjusting the variables associated with mood episodes, including age (per year), gender (female/male), residual mood symptoms (yes/no), multiple mood episodes within the past year before the baseline assessment (yes/no), total sleep time (abnormal sleep time [<6 or ≥9 h]/normal sleep time [≥6 and <9 h]), sleep efficiency (high/low, category based on the median value), and daytime illuminance (high/low) [11, 23–27]. We checked the multicollinearity among independent variables using the Spearman’s rank correlation coefficient. No serious multicollinearity was observed among any independent variable (*r*s < 0.5).

## Results

Among the 218 patients who participated in the present study, 19 were excluded because their baseline circadian activity rhythm data covered fewer than 3 days, and 10 were excluded because they were lost to follow-up within 12 months (4 were transferred to another clinic, 5 did not continuously attend the clinic visits, and 1 died from a physical illness). We analyzed the data from 189 participants who completed both the circadian activity rhythm data for at least 3 days and the 12-month follow-up. Table 1 presents the participants’ baseline characteristics, including their demographic and clinical characteristics, medication, and circadian activity rhythm parameters. The median age of the 189 participants was 44.0 (36.0–53.0) years, and 105 (55.6%) were female. The median (IQR) circadian activity rhythm level parameters were as follows: MESOR, 114.6 (82.7–151.9) counts/min; amplitude, 81.6 (59.5–116.3) counts/min; L5, 14.2 (8.1–23.7) counts/min; M10, 181.5 (133.2–240.1) counts/min; and relative amplitude, 0.85 (0.72–0.92). The median (IQR) circadian activity rhythm timing parameters were as follows: acrophase, 14:18 (13:15–15:22); L5 onset, 23:40 (22:31–24:43); and M10 onset, 8:01 (6:44–9:47).

**Table 1:** Baseline characteristics of the 189 participants.

Characteristics		Mood episode relapse during the follow-up		*P*
	All (*n* = 189)	Yes (*n* = 88)	No (*n* = 101)	
*Demographic characteristics*				
Age, years, median (IQR)	44.0 (36.0–53.0)	41.0 (33.0–48.7)	47.0 (39.0–55.5)	0.005
Gender, female, *n*	105 (55.6%)	53 (60.2%)	52 (51.1%)	0.228
Married, *n*	99 (52.4%)	43 (48.9%)	56 (55.4%)	0.366
Education (≥13 years), *n*	112 (59.3%)	44 (50.0%)	68 (67.3%)	0.016
Employed, *n*	76 (40.4%)	31 (35.6%)	45 (44.6%)	0.214
*Clinical characteristics*				
Type of bipolar disorder, bipolar disorder I, *n*	68 (36.0%)	32 (36.4%)	36 (35.6%)	0.918
Age at onset of bipolar disorder, years, median (IQR)	31.0 (22.2–39.0)	30.0 (22.0–36.0)	32.0 (24.0–42.0)	0.072
Duration of illness, years, median (IQR)	11.0 (6.2–16.0)	10.0 (6.0–15.0)	12.0 (7.0–17.5)	0.214
MADRS score, points, median (IQR)	8.0 (3.0–14.0)	11.0 (6.0–16.0)	6.0 (2.0–12.0)	0.001
YMRS score, points, median (IQR)	2.0 (0–5.0)	3.0 (0–5.0)	2.0 (0–4.0)	0.019
Residual mood symptoms, *n*	113 (59.8%)	64 (73.6%)	49 (49.0%)	0.001
Multiple mood episodes within 1 year before baseline assessment, *n*	61 (32.6%)	41 (46.6%)	20 (20.2%)	<0.001
Bedtime, clock time, median (IQR)	23:11 (22:11–24:16)	23:17 (22:16–24:30)	23:00 (22:08–24:04)	0.167
Rising time, clock time, median (IQR)	7:00 (6:12–7:48)	7:12 (6:15–8:20)	6:55 (6:06–7:33)	0.034
Total sleep time, min, median (IQR)	386.0 (334.6–434.3)	378.5 (329.0–452.1)	388.8 (336.3–432.3)	0.843
Sleep efficiency, %, median (IQR)	83.6 (78.1–87.8)	83.4 (76.2–87.2)	84.1 (79.3–88.5)	0.175
Daytime illuminance, lux, median (IQR)	224.2 (150.6–302.4)	201.8 (124.5–282.2)	250.9 (171.0–324.9)	0.019
*Medication*				
Lithium, *n*	76 (40.2%)	35 (39.8%)	41 (40.6%)	0.909
Anticonvulsants, *n*	108 (57.1%)	57 (64.8%)	51 (50.5%)	0.048
Antipsychotics, *n*	106 (56.1%)	46 (52.3%)	60 (59.4%)	0.324
Antidepressants, *n*	68 (36.0%)	30 (34.1%)	38 (37.6%)	0.614
Hypnotics, *n*	121 (64.0%)	62 (70.5%)	59 (58.4%)	0.085
*Circadian activity rhythm parameters, median (IQR)*				
Cosinor analysis				
MESOR, counts/min	114.6 (82.7–151.9)	100.2 (72.7–136.1)	128.0 (94.6–162.9)	0.001
Amplitude counts/min	81.6 (59.5–116.3)	74.1 (54.3–102.8)	93.0 (64.7–122.3)	0.005
Period, clock time	23:57 (23:43–24:12)	23:58 (23:48–24:16)	23:54 (23:41–24:08)	0.078
Acrophase, clock time	14:18 (13:15–15:22)	14:23 (13:23–15:40)	14:13 (13:06–15:12)	0.119
Nonparametric circadian rhythm analysis				
Interdaily stability	0.47 (0.37–0.58)	0.46 (0.32–0.56)	0.48 (0.38–0.58)	0.184
Intradaily variability	0.89 (0.72–1.08)	0.94 (0.72–1.09)	0.89 (0.72–1.05)	0.579
L5, counts/min	14.2 (8.1–23.7)	14.3 (8.5–25.5)	14.0 (7.8–21.6)	0.628
L5 onset, clock time	23:40 (22:31–24:43)	23:59 (22:33–25:08)	23:10 (22:26–24:32)	0.122
M10, counts/min	181.5 (133.2–240.1)	153.2 (118.8–218.7)	201.8 (145.0–255.8)	0.002
M10 onset, clock time	8:01 (6:44–9:47)	8:37 (7:18–10:16)	7:40 (6:26–9:22)	0.004
Relative amplitude	0.85 (0.72–0.92)	0.81 (0.70–0.91)	0.86 (0.76–0.92)	0.047

Data are expressed as median (interquartile range) or number (%).

*MADRS* Montgomery–Åsberg Depression Rating Scale, *YMRS* Young Mania Rating Scale, *MESOR* midline-estimating statistic of rhythm, *L5* least active continuous 5-h period, *M10* most active continuous 10-h period.

**Fig. 1:**
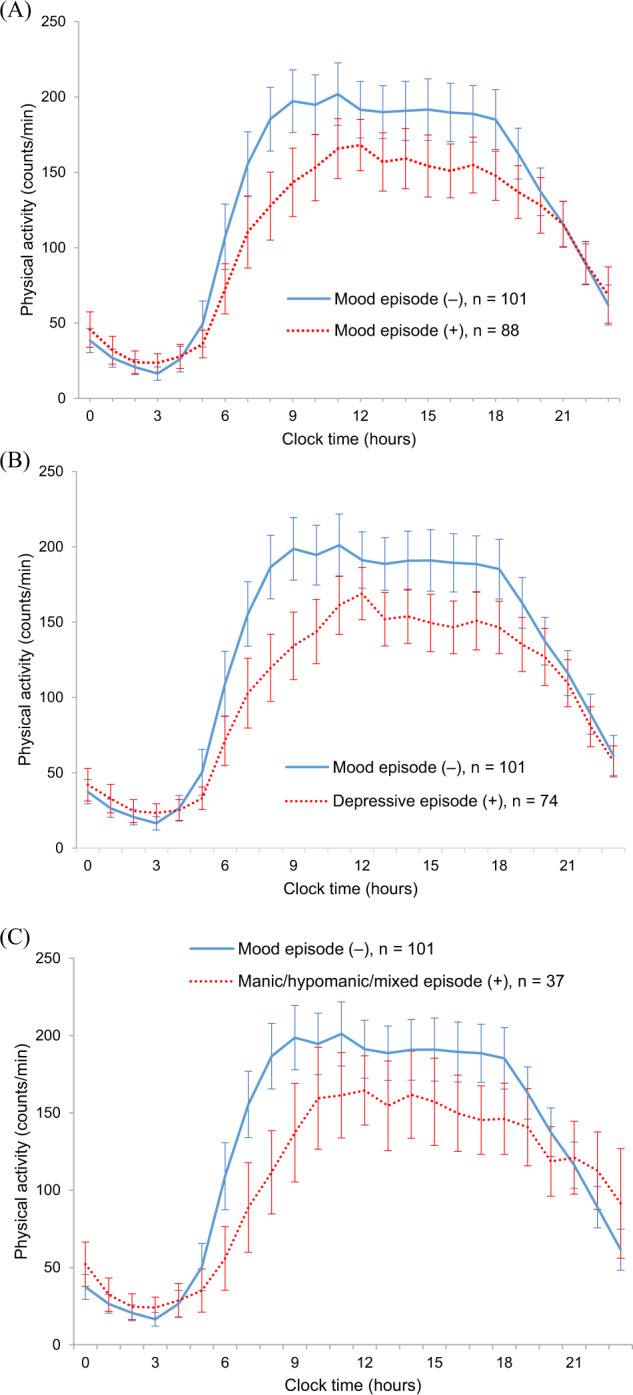
Line graph of mean physical activity counts from midnight to 23:00 for 189 patients with bipolar disorder. The solid blue line indicates participants who did not experience mood episodes during the 12-month follow-up. The dotted red line indicates the participants who experienced mood (**A**), depressive (**B**), or manic/hypomanic/mixed (**C**) episodes during the 12-month follow-up. Physical activity counts are plotted for each hour. Error bar presents 95% confidence interval.

Of the 189 participants, 88 (46%) experienced mood episodes during the 12-month follow-up period. Of these, 74 (39%) experienced depressive episodes, and 37 (19%) experienced manic, hypomanic, or mixed episodes. Of these, 6 (3%) experienced manic episodes, 26 (13%) experienced hypomanic episodes, and 5 (2%) experienced mixed episodes. Several participants experienced multiple mood episodes. The median visit interval during follow-up was 3.4 (2.2–4.3) weeks. The median period from baseline assessment to subsequent mood episode relapses was 5.5 (2.0–8.7) months. Figure 1 presents a visualization of the mean physical activity counts of the participants in terms of mood episode experience during the 12-month follow-up.

Survival analysis using the Kaplan–Meier method indicated that MESOR, amplitude, L5 onset, M10, and M10 onset were significantly associated with mood episode relapses (Supplemental Fig. 1). The Cox proportional hazards models revealed that a robust circadian activity rhythm, including higher MESOR, amplitude, M10, and relative amplitude, was significantly associated with a decrease in mood episode relapses (crude model; Table 2). The association remained significant in the adjusted model for age and gender (adjusted model 1; Table 2). After adjusting for age, gender, residual mood symptoms, multiple mood episodes within 1 year before the baseline assessment, total sleep time, sleep efficiency, and daytime illuminance, we found that higher MESOR, amplitude, and M10 showed a significant decrease in mood episode relapses (adjusted model 2; Table 2). Although a later timing of the circadian activity rhythm, including acrophase and M10 onset, was significantly associated with an increase in mood episode relapses (crude model; Table 2), this association was not significant after adjusting for all covariates (adjusted model 2; Table 2).

**Table 2:** Cox proportional hazards analysis for mood episode relapse associated with circadian activity rhythm parameters.

Circadian activity rhythm parameters	Crude model		Adjusted model 1		Adjusted model 2	
	HR (95% CI)	*P*	HR (95% CI)	*P*	HR (95% CI)	*P*
Cosinor analysis						
MESOR, per counts/min	0.994 (0.989–0.998)	0.003	0.993 (0.988–0.997)	0.002	0.993 (0.988–0.997)	0.002
Amplitude, per counts/min	0.993 (0.988–0.998)	0.006	0.992 (0.987–0.998)	0.006	0.994 (0.988–0.999)	0.022
Period, per hour	1.548 (0.969–2.475)	0.068	1.415 (0.887–2.257)	0.145	1.267 (0.796–2.015)	0.318
Acrophase, per hour	1.117 (1.014–1.231)	0.025	1.067 (0.959–1.186)	0.234	1.040 (0.932–1.161)	0.480
Nonparametric circadian rhythm analysis						
Interdaily stability	0.336 (0.072–1.562)	0.163	0.435 (0.090–2.101)	0.300	0.810 (0.159–4.113)	0.799
Intradaily variability	1.393 (0.669–2.901)	0.376	1.513 (0.705–3.248)	0.288	1.436 (0.648–3.180)	0.372
L5, per log counts/min	1.050 (0.804–1.370)	0.721	1.049 (0.796–1.382)	0.734	0.799 (0.566–1.127)	0.201
L5 onset, per hour	1.049 (0.961–1.145)	0.284	1.020 (0.934–1.115)	0.658	0.993 (0.908–1.085)	0.872
M10, per counts/min	0.996 (0.993–0.999)	0.004	0.996 (0.993–0.998)	0.003	0.996 (0.993–0.999)	0.007
M10 onset, per hour	1.128 (1.043–1.219)	0.003	1.104 (1.019–1.196)	0.016	1.079 (0.993–1.173)	0.073
Relative amplitude	0.243 (0.062–0.949)	0.042	0.202 (0.050–0.822)	0.025	0.435 (0.075–2.537)	0.355

The L5 parameter was analyzed after natural logarithmic transformation. Model 1 was adjusted for age and gender. Model 2 was adjusted for age, gender, residual mood symptoms, multiple mood episodes within 1 year before baseline assessment, total sleep time, sleep efficiency, and daytime illuminance.

*HR* hazard ratio, *CI* confidence interval, *MESOR* midline-estimating statistic of rhythm, *L5* least active continuous 5-h period, *M10* most active continuous 10-h period.

Regarding the association between circadian activity rhythm parameters and subsequent depressive episodes, we found that a robust circadian activity rhythm, including higher MESOR, amplitude, and M10, was significantly associated with a decrease in the relapse of depressive episodes (crude model; Table 3). We observed similar significance in the adjusted models 1 and 2 (Table 3). A later timing of the circadian activity rhythm, including acrophase and M10 onset, was significantly associated with an increase in the relapse of depressive episodes (crude model; Table 3). The association between M10 onset and the relapse of depressive episodes remained significant in the adjusted models 1 and 2 (Table 3). Although a longer period was significantly associated with an increase in the relapse of depressive episodes (crude model; Table 3), this association was not significant in the adjusted models 1 and 2 (Table 3).

**Table 3:** Cox proportional hazards analysis for depressive episode relapse associated with circadian activity rhythm parameters.

Circadian activity rhythm parameters	Crude model		Adjusted model 1		Adjusted model 2	
	HR (95% CI)	*P*	HR (95% CI)	*P*	HR (95% CI)	*P*
Cosinor analysis						
MESOR, per counts/min	0.992 (0.987–0.997)	0.001	0.991 (0.986–0.996)	0.001	0.990 (0.985–0.995)	<0.001
Amplitude, per counts/min	0.992 (0.986–0.998)	0.007	0.992 (0.986–0.998)	0.009	0.993 (0.987–0.999)	0.019
Period, per hour	1.745 (1.062–2.869)	0.028	1.631 (0.993–2.680)	0.053	1.528 (0.915–2.553)	0.105
Acrophase, per hour	1.117 (1.002–1.244)	0.045	1.077 (0.957–1.212)	0.221	1.068 (0.945–1.208)	0.292
Nonparametric circadian rhythm analysis						
Interdaily stability	0.348 (0.067–1.799)	0.348	0.439 (0.083–2.333)	0.334	0.605 (0.107–3.421)	0.570
Intradaily variability	1.220 (0.553–2.690)	0.622	1.292 (0.572–2.916)	0.538	1.169 (0.506–2.702)	0.714
L5, per log counts/min	0.956 (0.717–1.273)	0.756	0.947 (0.705–1.271)	0.716	0.645 (0.449–0.927)	0.018
L5 onset, per hour	1.032 (0.934–1.140)	0.539	1.007 (0.910–1.115)	0.889	0.991 (0.893–1.100)	0.867
M10, per counts/min	0.995 (0.992–0.998)	0.002	0.995 (0.992–0.998)	0.002	0.995 (0.991–0.998)	0.002
M10 onset, per hour	1.114 (1.048–1.242)	0.002	1.120 (1.027–1.223)	0.011	1.109 (1.001–1.215)	0.028
Relative amplitude	0.320 (0.069–1.491)	0.147	0.305 (0.064–1.457)	0.137	0.887 (0.118–6.664)	0.907

The L5 parameter was analyzed after natural logarithmic transformation. Model 1 was adjusted for age and gender. Model 2 was adjusted for age, gender, residual mood symptoms, multiple mood episodes within 1 year before baseline assessment, total sleep time, sleep efficiency, and daytime illuminance.

*HR* hazard ratio, *CI* confidence interval, *MESOR* midline-estimating statistic of rhythm, *L5* least active continuous 5-h period, *M10* most active continuous 10-h period.

Although a later timing of the circadian activity rhythm, including acrophase, L5 onset, and M10 onset, was significantly associated with an increase in the relapse of manic/hypomanic/mixed episodes (crude model; Table 4), this association was not significant in the adjusted models 1 and 2 (Table 4), in which a higher intradaily variability was significantly associated with an increase in the relapse of manic/hypomanic/mixed episodes (Table 4).

**Table 4:** Cox proportional hazards analysis for relapse of manic/hypomanic/mixed episodes associated with circadian activity rhythm parameters.

Circadian activity rhythm parameters	Crude model		Adjusted model 1		Adjusted model 2	
	HR (95% CI)	*P*	HR (95% CI)	*P*	HR (95% CI)	*P*
Cosinor analysis						
MESOR, per counts/min	0.994 (0.987–1.001)	0.078	0.993 (0.985–1.000)	0.055	0.993 (0.986–1.001)	0.080
Amplitude, per counts/min	0.993 (0.985–1.001)	0.104	0.992 (0.983–1.001)	0.084	0.994 (0.985–1.003)	0.198
Period, per hour	1.034 (0.490–2.183)	0.930	0.901 (0.433–1.873)	0.780	0.846 (0.415–1.726)	0.646
Acrophase, per hour	1.156 (1.009–1.324)	0.036	1.079 (0.929–1.253)	0.318	1.053 (0.900–1.232)	0.519
Nonparametric circadian rhythm analysis						
Interdaily stability	0.348 (0.035–3.449)	0.367	0.417 (0.039–4.439)	0.469	0.951 (0.084–10.746)	0.967
Intradaily variability	2.869 (0.999–8.242)	0.050	3.726 (1.224–11.338)	0.021	3.391 (1.044–11.016)	0.042
L5, per log counts/min	1.192 (0.785–1.811)	0.410	1.201 (0.774–1.864)	0.413	1.085 (0.623–1.891)	0.773
L5 onset, per hour	1.149 (1.039–1.271)	0.007	1.109 (0.999–1.230)	0.053	1.081 (0.969–1.206)	0.162
M10, per counts/min	0.996 (0.992–1.000)	0.081	0.995 (0.991–1.000)	0.056	0.996 (0.931–1.001)	0.115
M10 onset, per hour	1.132 (1.008–1.272)	0.036	1.091 (0.970–1.228)	0.147	1.064 (0.938–1.208)	0.333
Relative amplitude	0.262 (0.037–1.872)	0.182	0.182 (0.023–1.449)	0.108	0.296 (0.022–4.046)	0.362

The L5 parameter was analyzed after natural logarithmic transformation. Model 1 was adjusted for age and gender. Model 2 was adjusted for age, gender, residual mood symptoms, multiple mood episodes within 1 year before baseline assessment, total sleep time, sleep efficiency, and daytime illuminance.

*HR* hazard ratio, *CI* confidence interval, *MESOR* midline-estimating statistic of rhythm, *L5* least active continuous 5-h period, *M10* most active continuous 10-h period.

We conducted an additional cosine analysis with the circadian activity rhythm periods of all participants as 24 h. The results were consistent with those of the cosinor analysis, which assigned the individual circadian activity rhythm periods for each participant (Supplemental Tables 1–3).

## Discussion

To our knowledge, this is the first prospective cohort study to report the association between circadian activity rhythms objectively measured using a wrist-worn accelerometer and subsequent mood episodes in bipolar disorder. We found that a robust circadian activity rhythm was significantly associated with a decrease in mood episode relapses, mainly depressive episodes. We also found that a later timing of the circadian activity rhythm was a significant factor in increasing the relapse of depressive episodes. These associations were independent of the confounding factors associated with mood episodes, including age, gender, residual mood symptoms, multiple mood episodes within the past year before the baseline assessment, total sleep time, sleep efficiency, and daytime illuminance.

Our results indicate that dampened circadian activity rhythm, including lower MESOR, amplitude, and M10, was significantly associated with an increase in mood episode relapses. A prospective 18-month study of 80 patients with bipolar disorder reported an association between lower physical activity measured using the International Physical Activity Questionnaire and increased mood episodes [9]. Our findings support those of the previous study and further suggest that physical activity levels are mainly associated with the depressive episode relapses. A systematic review of 15,587 patients with bipolar disorder reported that physical activity was associated with less depressive symptoms [8]. A prospective 11-year cohort study of 33,908 adults demonstrated that engaging in at least 1 h of physical activity each week could prevent 12% of future cases of depression [28]. Furthermore, the evidence base for exercise as a therapy for current depression is being established [29]. Increased physical activity levels might therefore prevent mood episode relapses, mainly depressive episodes.

We also observed that a later timing of the circadian activity rhythm was significantly associated with an increase in depressive episode relapses. A prospective 48-week study of 104 patients with bipolar disorder reported that the comorbidity of circadian rhythm sleep–wake disorders, mainly delayed sleep–wake phase disorder, was significantly associated with the time to relapse of mood episodes [11]. A systematic review of clinical studies in bipolar disorder suggested that the depressive period was more frequently associated with circadian alterations than the euthymic and hypomanic periods [30]. Our results showed a significant association between delayed M10 onset and increased depressive episode relapses. Our findings and those of previous studies therefore indicate that circadian disruption, especially delayed timing, is associated with depressive episode relapses. We also found that higher intradaily variability was significantly associated with increased relapse of manic/hypomanic/mixed episodes. Studies have reported that the manic state is associated with circadian abnormalities, including advanced circadian phases and increased melatonin secretion [31, 32]. Furthermore, interpersonal and social rhythm therapy, which is a psychosocial therapy targeted at stabilizing the daily rhythms combined with interpersonal psychotherapy, has been reported to be effective for relapse prevention of new affective episodes in patients with bipolar disorder [33]. Thus, circadian rhythm abnormalities might affect the relapse of manic, hypomanic, and mixed episodes. Further investigations are necessary to clarify the association between the timing of physical activity and subsequent mood episodes.

Our findings suggest adjunctive treatment to prevent recurrence or relapse of mood episodes in patients with bipolar disorder. Although pharmacotherapy is an effective treatment for preventing mood episode relapse in bipolar disorder, the therapy causes various adverse effects, including liver and renal disorders, extrapyramidal symptoms, akathisia, sedation, and weight gain [3]. As a result, patients with bipolar disorder can face the dual risk of mental and physical problems. In contrast, exercise is reported to have a positive effect not only on mental problems, including mood symptoms and relapse of mood episodes, but also on physical problems, such as obesity, hypertension, and cholesterol [8, 9, 34, 35]. Furthermore, we found that an increase in M10 from 133 to 240 counts/min (25th to 75th percentiles) was associated with a 42.8% decrease in mood episode relapses. Given that many patients with bipolar disorder are reported to spend the majority of their time being sedentary [36], increased physical activity such as exercise might be useful as an adjunct therapy to improve both mental and physical problems in patients with bipolar disorder.

Our findings may also help to clarify the mechanisms underlying the effect of lithium for relapses of mood episodes. Lithium is the first-line treatment for preventing relapses and recurrences of mood episodes in bipolar disorder [37]. However, its mechanism of action remains unknown. Previous studies have suggested that lithium was associated with the period, phase, and amplitude [38–40]. Our results found that circadian activity rhythm was significantly associated with mood episode relapses. Therefore, this evidence suggests that lithium may prevent relapses and recurrences of mood episodes through modulation of circadian activity rhythms.

To date, the mechanisms underlying the association between physical activity and mood episode relapses in bipolar disorder remain unclear; however, several potential mechanisms have been reported. A previous study reported lower activity and later timing of sleep offset and rest offset in individuals with euthymic bipolar disorder compared with their relatives without bipolar disorder and heritability for phenotypes assaying multiple facets of sleep and activity [41]. Moreover, another study reported that a lower relative amplitude was associated with an increased risk of lifetime bipolar disorder [42]. Genetic factors might therefore contribute to circadian activity rhythm phenotypes in bipolar disorder, resulting in mood episode relapses. Environmental factors, such as lifestyle, are another possible mechanism. Physical activity has been reported to alter the progression of mood disorder neuropathology by optimizing neurotransmission function, brain-derived neurotrophic factor, endorphins, and cortisol [43, 44]. Furthermore, regular physical activity improves neurogenesis, immune–inflammatory function, stress regulation, antioxidant defense, circadian rhythms, and epigenetic modifications [43, 44]. Physical activity is therefore involved in numerous mechanisms; however, it is unclear which of these mechanisms contribute to mood episode relapses. Further studies are warranted to determine whether any of these processes mediate between physical activity and mood episode relapses in bipolar disorder.

Our study has several notable strengths and limitations. Its strengths include its prospective cohort, the objectively measured circadian activity rhythms, and the two different circadian activity rhythm analysis methods. The cosinor analysis is a parametric analysis, which fits a symmetric cosinor curve into the circadian rhythm. However, the circadian rhythm of daily activity is not symmetric, with a shorter night curve and a longer day curve (Fig. 1). Our study therefore complemented this limitation with a nonparametric circadian rhythm analysis.

The study’s limitations include, first, the fact that the study participants were not randomly selected, which might have affected the results with a selection bias. Second, the psychiatric medications at the baseline assessment were not adjusted. It is possible that these medications might have produced sedation in the patients with bipolar disorder and might have affected their circadian activity rhythm parameters and clinical course. Third, due to the naturalistic observational study design, the therapy during follow-up was not controlled in this study and did not necessarily follow specific contemporary practice guidelines. In addition, drug adherence was not investigated during the follow-up visits. The results might have been affected by the pharmacotherapy and psychotherapy. Fourth, each participant’s psychiatrist assessed the participant’s mood episodes within daily practices and were not instructed to systematically record the mood episodes according to DSM-5 criteria. The mood episodes might therefore not have been properly assessed. However, the proportion of mood episodes in our study (45%) was similar to the results of a meta-analysis on the risk of subsequent mood episodes in bipolar disorder (44% recurrence within 1 year) [1]. Furthermore, the median visit interval for the follow-up was 3.4 weeks, which indicates that the psychiatrists assessed mood episodes at least 12 times from the baseline assessment to 1 year. Therefore, the mood episode assessment employed in the present study might be acceptable. Finally, the circadian activity rhythm parameters were measured only at 7 days, suggesting that the measurement period might have been too short. In addition, there was no evidence that these data indicated a habitual physical activity pattern across the 12 consecutive months. Therefore, circadian rhythm parameters at the time of mood episode relapses may have differed from those at the time of the baseline assessment. Although we speculate that 7 days provided an approximate representation of an individual’s behavior pattern, given that the period includes weekdays and the weekend, further investigations using long-term measurements of physical activity might reveal more appropriate associations between physical activity and mood episode relapses.

In conclusion, our study showed that the level and timing of physical activity were significantly and independently associated with mood episode relapses, mainly depressive episodes, in patients with bipolar disorder. Further large and controlled studies are warranted to establish the association.

## Supplementary information

Supplemental Figure 1

Supplemental table 1

Supplemental table 2

Supplemental table 3
