# Supplementary material for: Association between circadian activity rhythms and mood episode relapse in bipolar disorder: a 12-month prospective cohort study
Source: Transl Psychiatry. 2021 Oct 13;11:525. doi: 10.1038/s41398-021-01652-9 (PMC8514471; doi:10.1038/s41398-021-01652-9)
Supplement: Supplementary file 1 — Supplemental Figure 1 [file 41398_2021_1652_MOESM1_ESM.pdf]

Supplemental Figure 1. Kaplan–Meier survival curves for time to mood episode relapse associated with circadian activity rhythm parameters in 189 patients with bipolar disorder

Participants were divided into two groups (category based on the median value) according to each circadian activity rhythm parameter, including midline-estimating statistic of rhythm (A), amplitude (B), period (C), acrophase (D), interdaily stability (E), intradaily variability (F), least active continuous 5-h period (G), least active continuous 5-h period onset (H), most active continuous 10-h period (I), most active continuous 10-h period (J), relative amplitude (K).

(A)

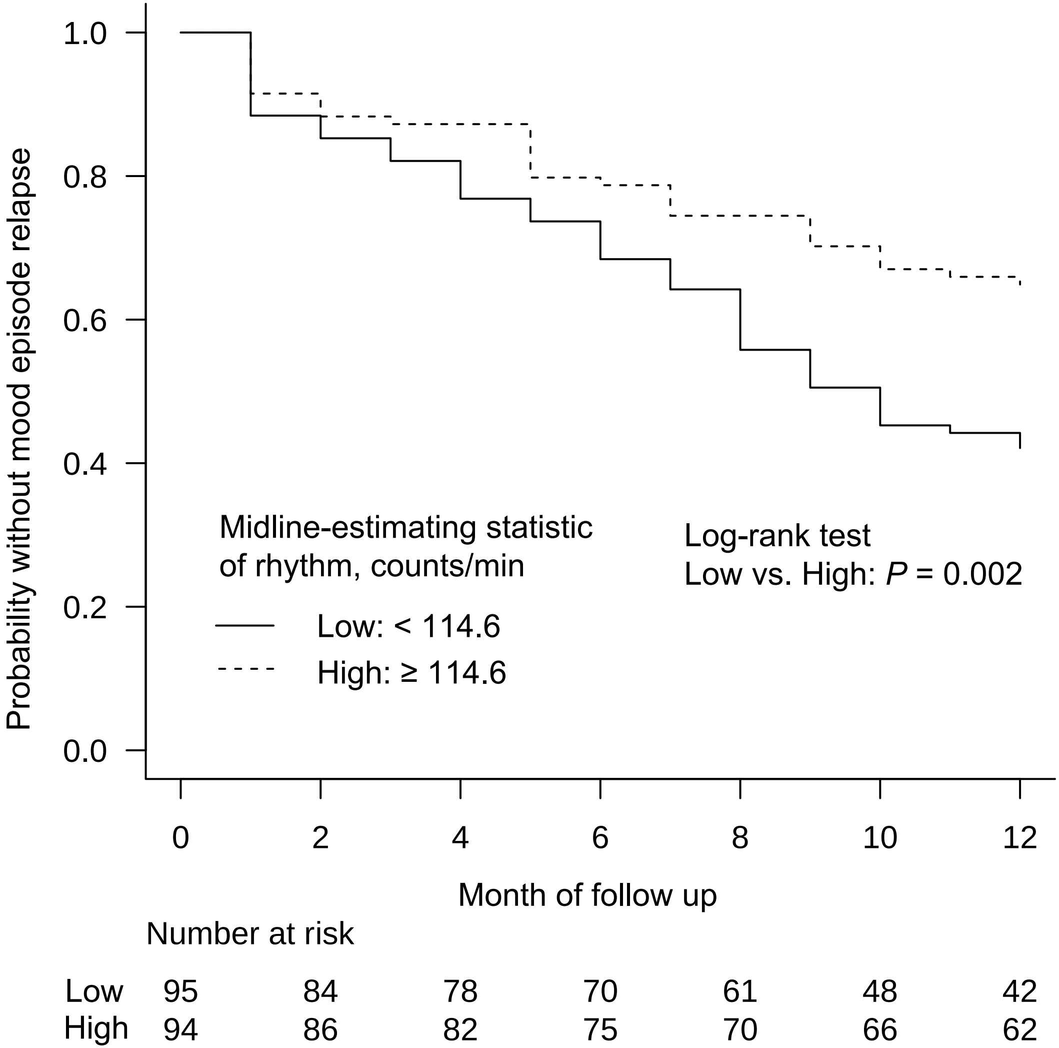
Probability without mood episode relapse
1.0
0.8
0.6
0.4
0.2
0.0
Midline-estimating statistic
of rhythm, counts/min
Low: < 114.6
High: ≥ 114.6
Log-rank test
Low vs. High: *P* = 0.002
0
2
4
6
8
10
12
Month of follow up
Number at risk
Low 95 84 78 70 61 48 42
High 94 86 82 75 70 66 62

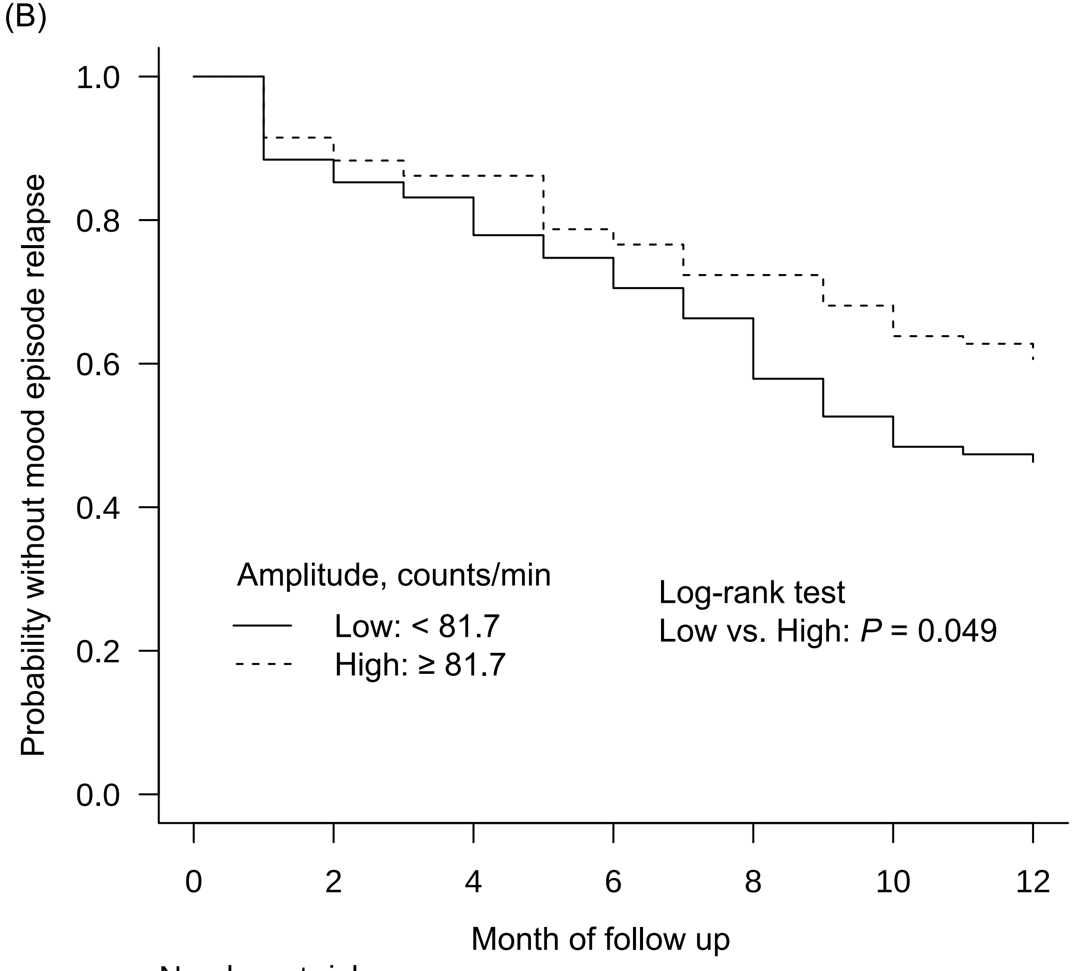

Number at risk

	0	2	4	6	8	10	12
Low	95	84	79	71	63	50	45
High	94	86	81	74	68	64	59

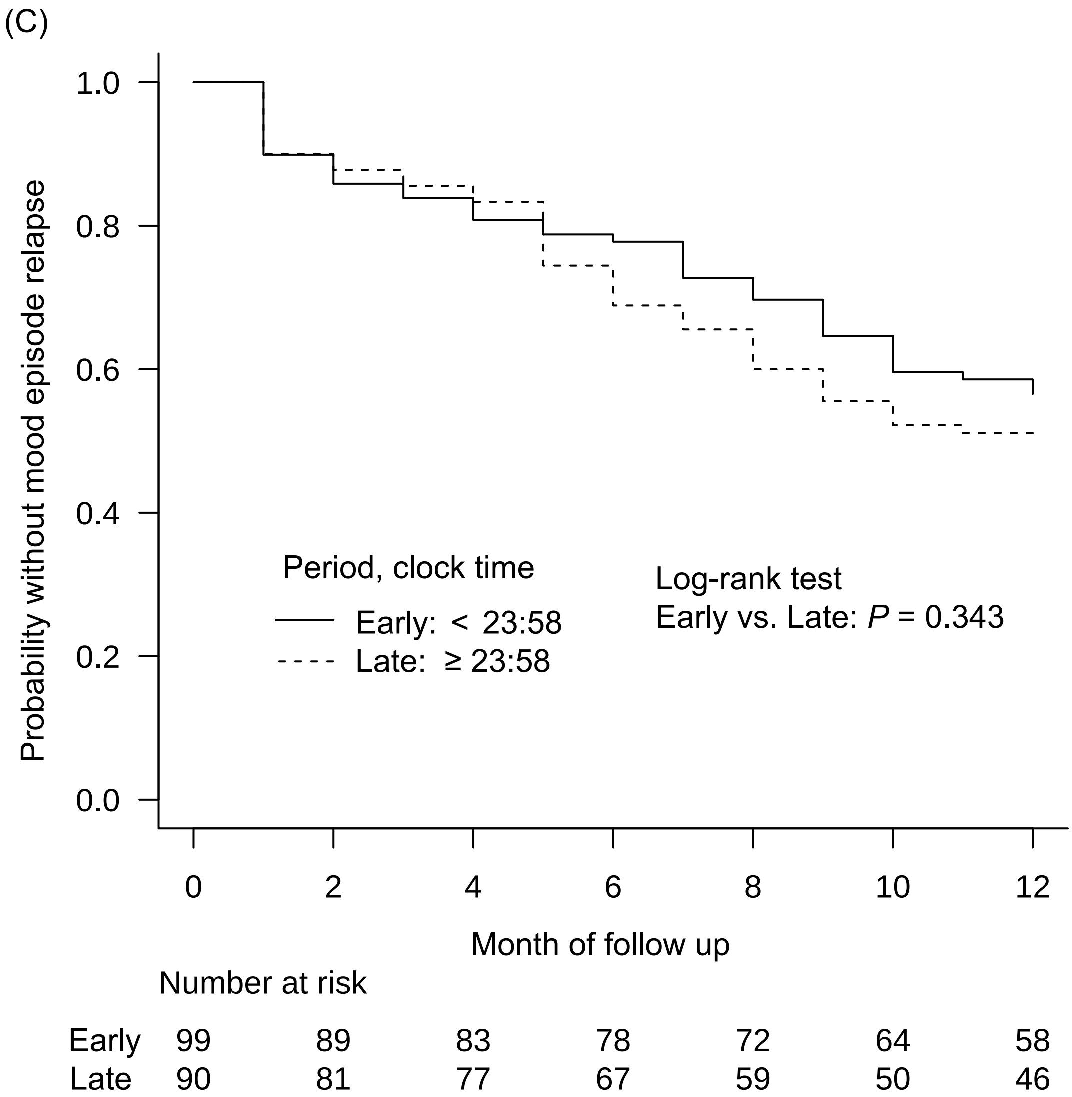
(C)
Probability without mood episode relapse
1.0
0.8
0.6
0.4
0.2
0.0
Period, clock time
Early: < 23:58
Late: ≥ 23:58
Log-rank test
Early vs. Late: P = 0.343
0
2
4
6
8
10
12
Month of follow up
Number at risk
Early 99 89 83 78 72 64 58
Late 90 81 77 67 59 50 46

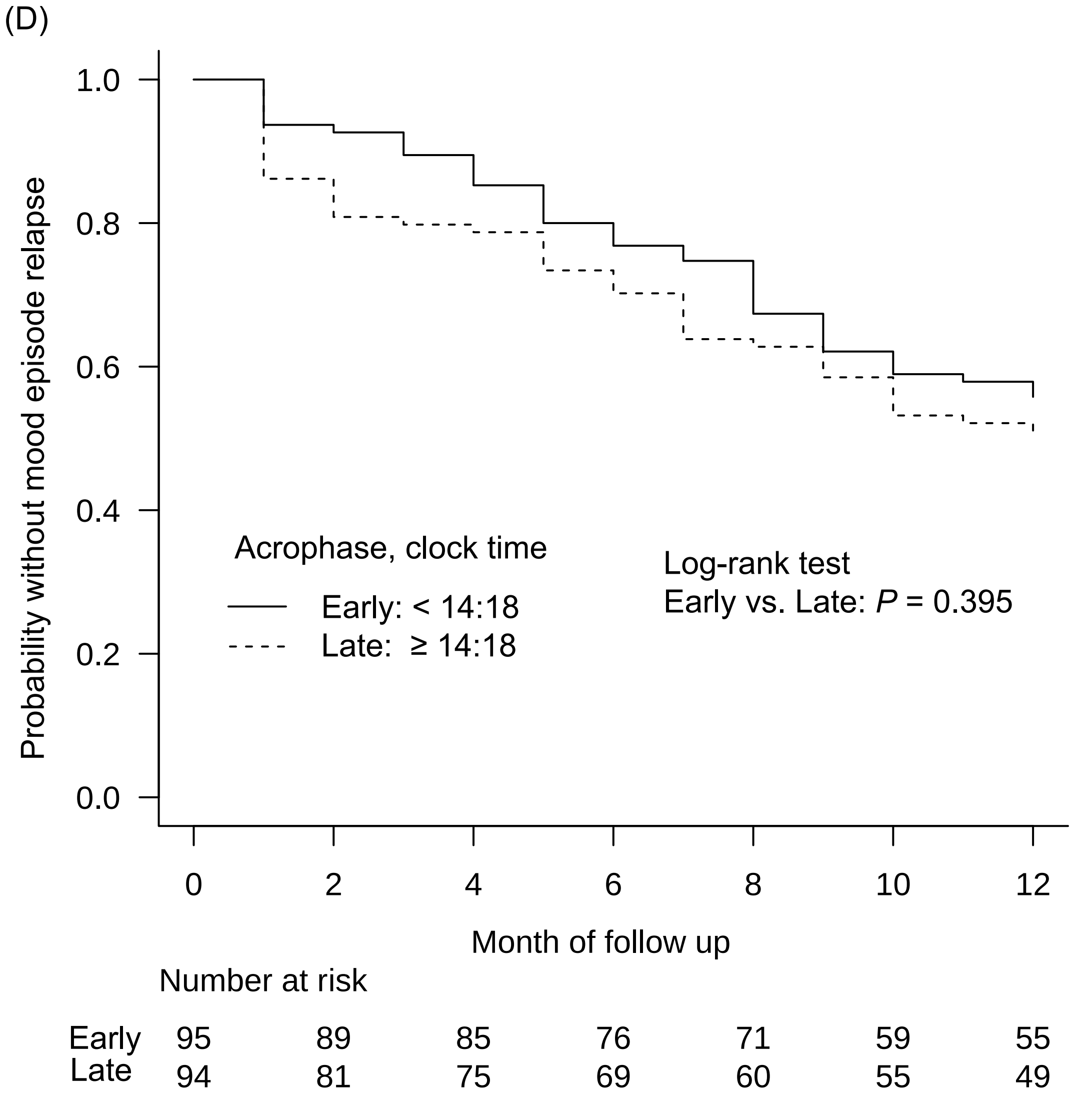
(D)
Probability without mood episode relapse
1.0
0.8
0.6
0.4
0.2
0.0
Acrophase, clock time
Early: < 14:18
Late: ≥ 14:18
Log-rank test
Early vs. Late: P = 0.395
0
2
4
6
8
10
12
Month of follow up
Number at risk
Early 95 89 85 76 71 59 55
Late 94 81 75 69 60 55 49

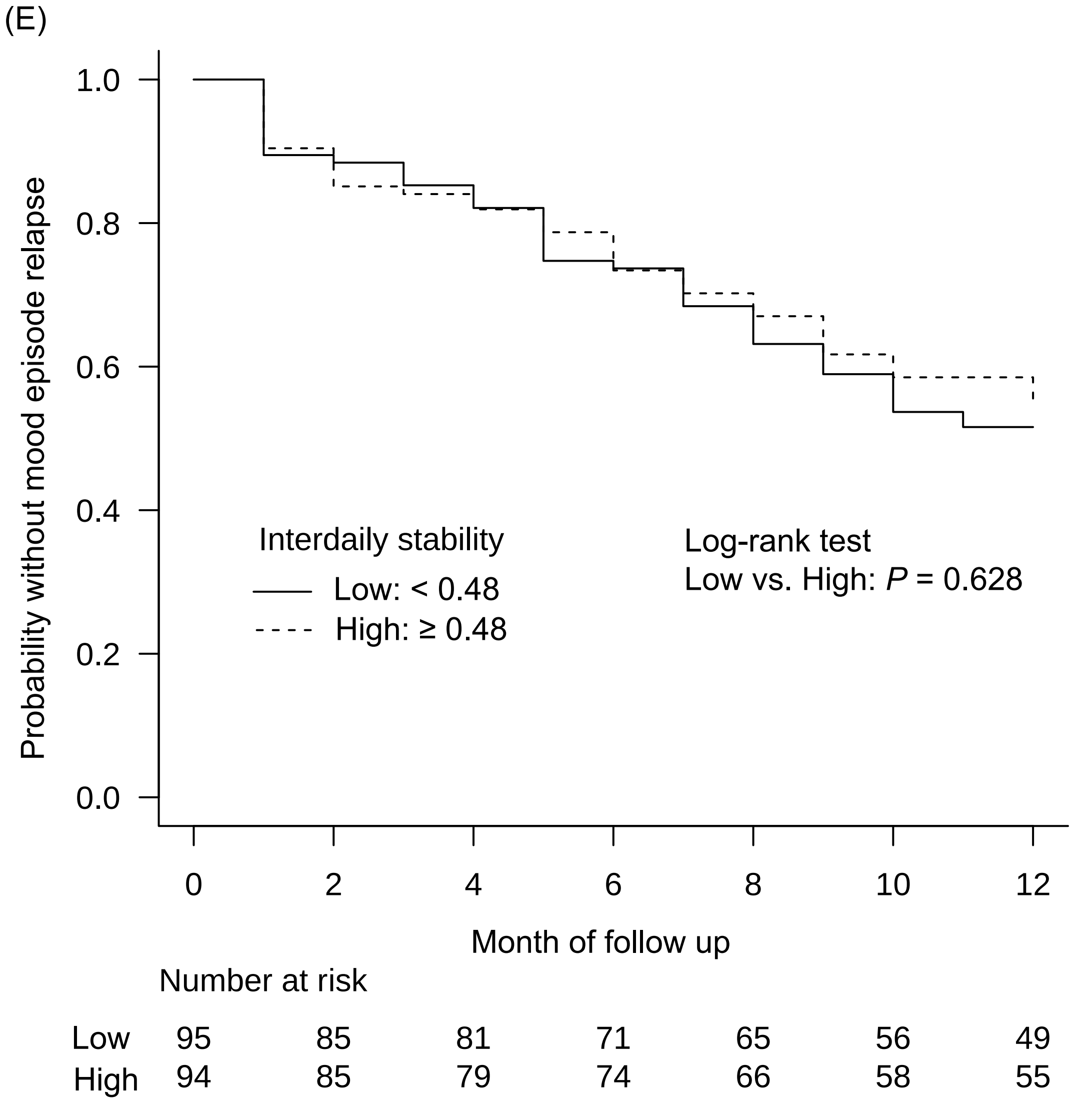
(E)
Probability without mood episode relapse
1.0
0.8
0.6
0.4
0.2
0.0
Interdaily stability
Low: < 0.48
High: ≥ 0.48
Log-rank test
Low vs. High: P = 0.628
0
2
4
6
8
10
12
Month of follow up
Number at risk
Low 95 85 81 71 65 56 49
High 94 85 79 74 66 58 55

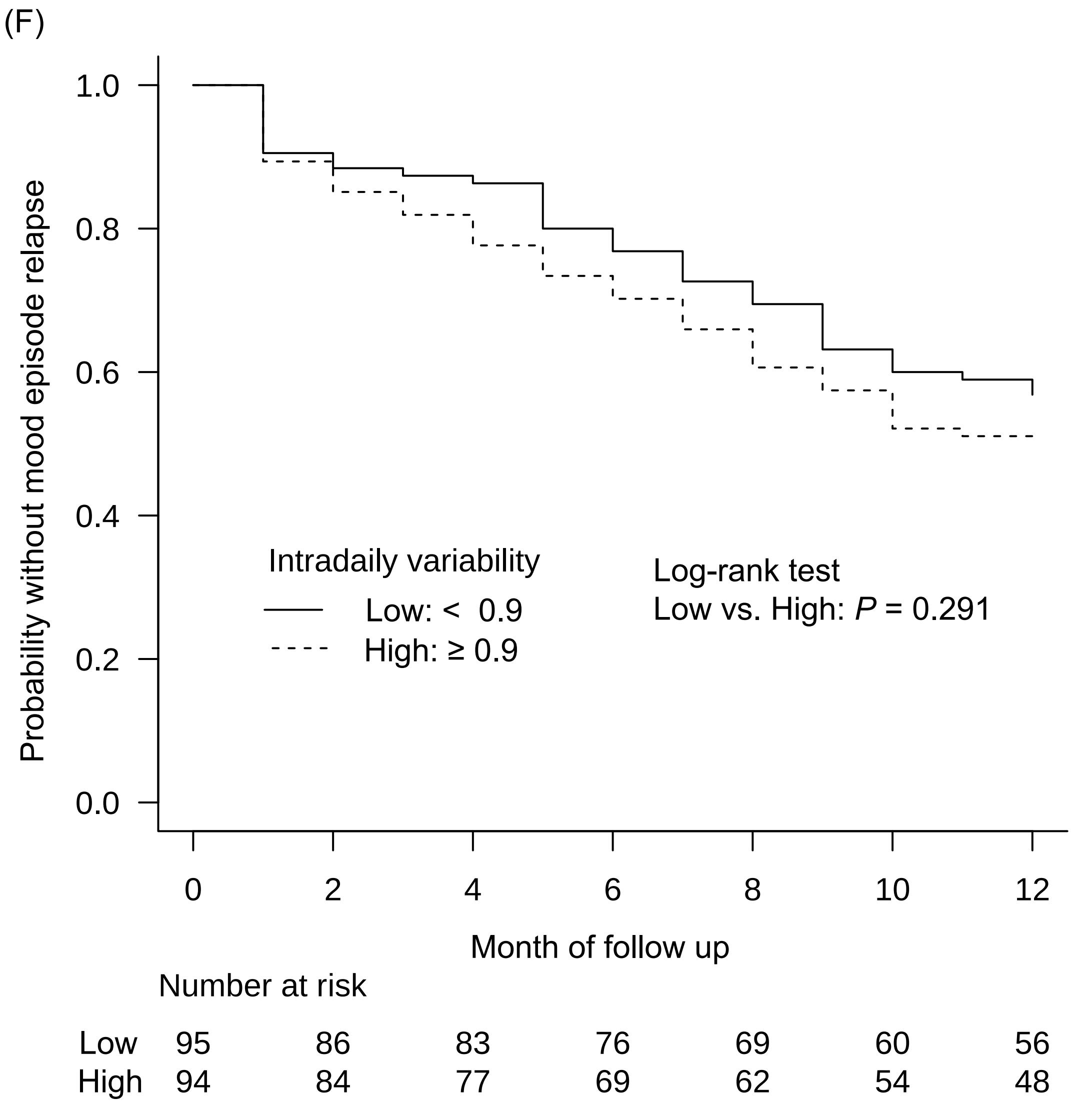

(F)
Probability without mood episode relapse
1.0
0.8
0.6
0.4
0.2
0.0
Intradaily variability
Low: < 0.9
High: ≥ 0.9
Log-rank test
Low vs. High: P = 0.291
0
2
4
6
8
10
12
Month of follow up
Number at risk
Low 95 86 83 76 69 60 56
High 94 84 77 69 62 54 48

(G)

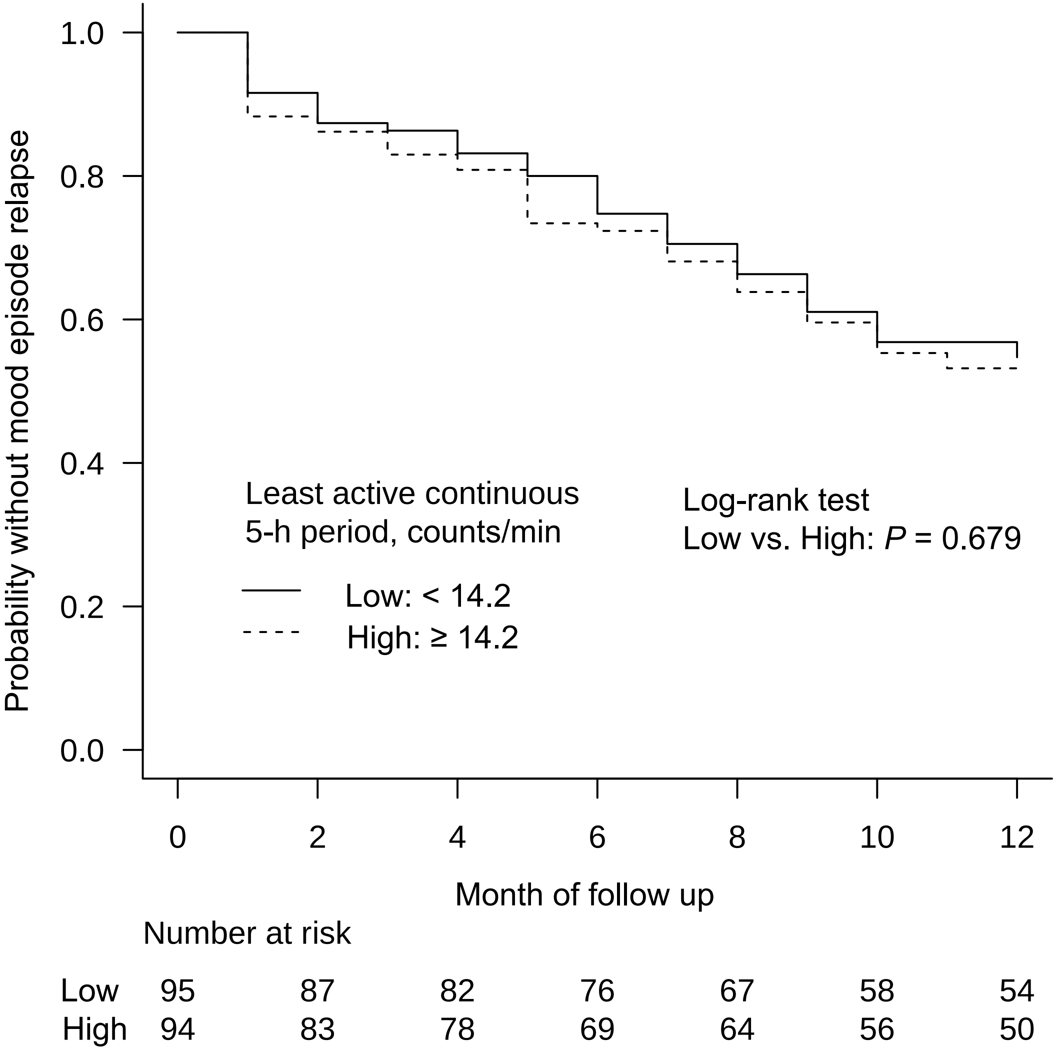
Probability without mood episode relapse
1.0
0.8
0.6
0.4
0.2
0.0
Least active continuous
5-h period, counts/min
Low: < 14.2
High: ≥ 14.2
Log-rank test
Low vs. High: P = 0.679
0
2
4
6
8
10
12
Month of follow up
Number at risk
Low 95 87 82 76 67 58 54
High 94 83 78 69 64 56 50

(H)

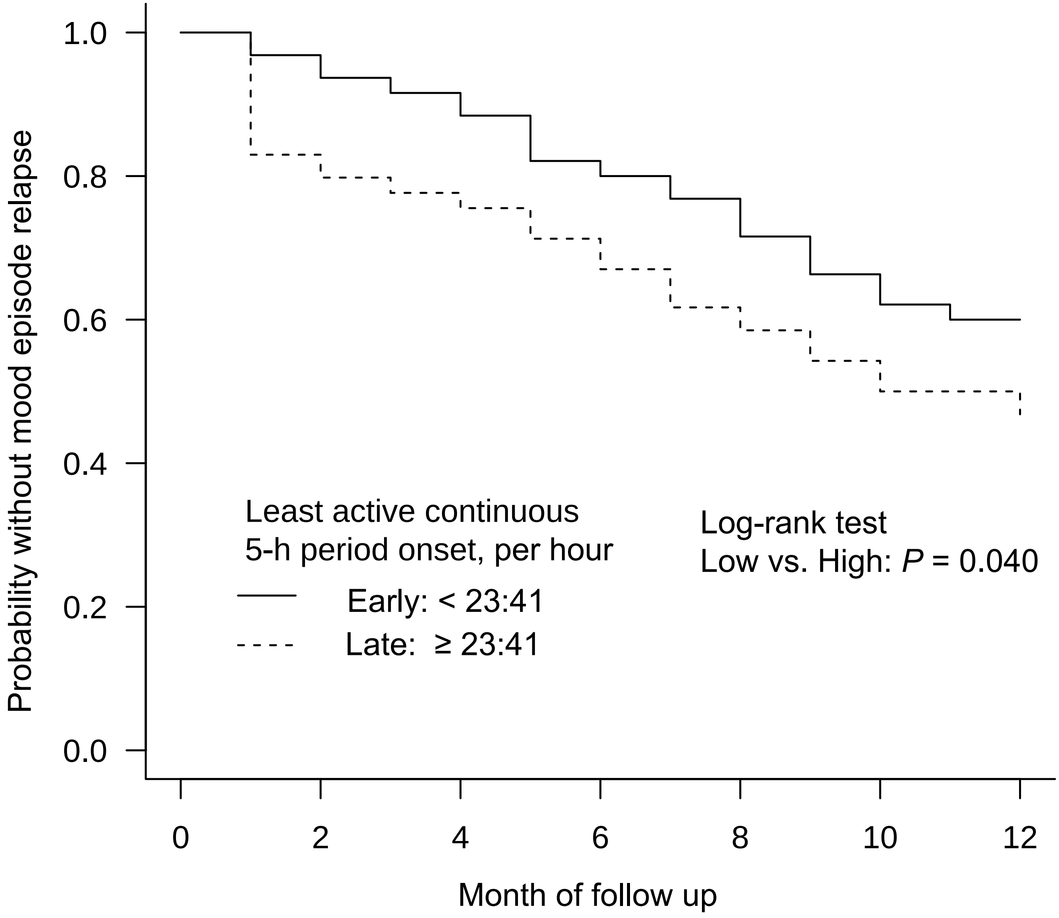

Number at risk

	0	2	4	6	8	10	12
Early	95	92	87	78	73	63	57
Late	94	78	73	67	58	51	47

(I)

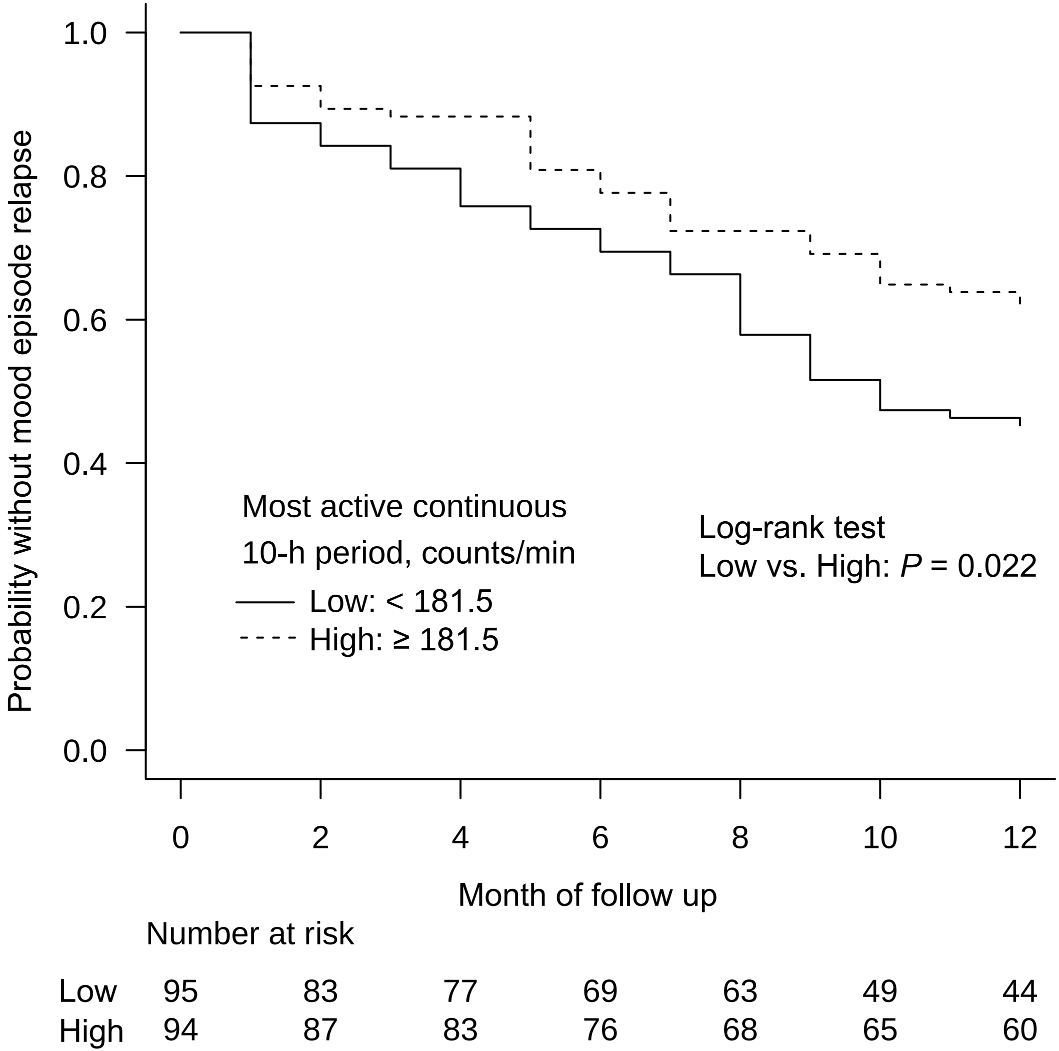

Probability without mood episode relapse
1.0
0.8
0.6
0.4
0.2
0.0
Most active continuous
10-h period, counts/min
Low: < 181.5
High: ≥ 181.5
Log-rank test
Low vs. High: *P* = 0.022
0
2
4
6
8
10
12
Month of follow up
Number at risk
Low 95 83 77 69 63 49 44
High 94 87 83 76 68 65 60

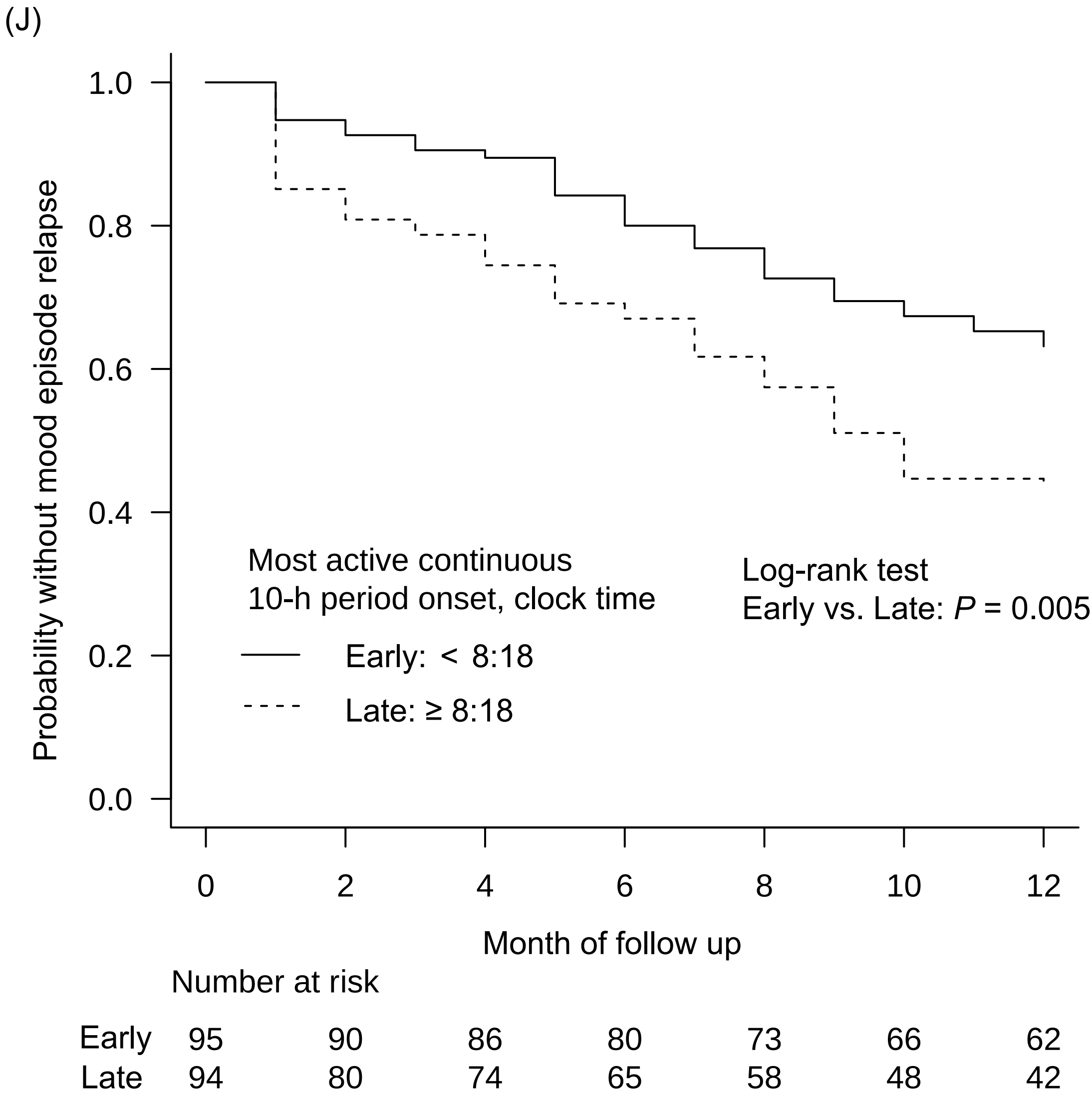
(J)
Probability without mood episode relapse
1.0
0.8
0.6
0.4
0.2
0.0
Most active continuous
10-h period onset, clock time
Early: < 8:18
Late: ≥ 8:18
Log-rank test
Early vs. Late: P = 0.005
0
2
4
6
8
10
12
Month of follow up
Number at risk
Early 95 90 86 80 73 66 62
Late 94 80 74 65 58 48 42

(K)

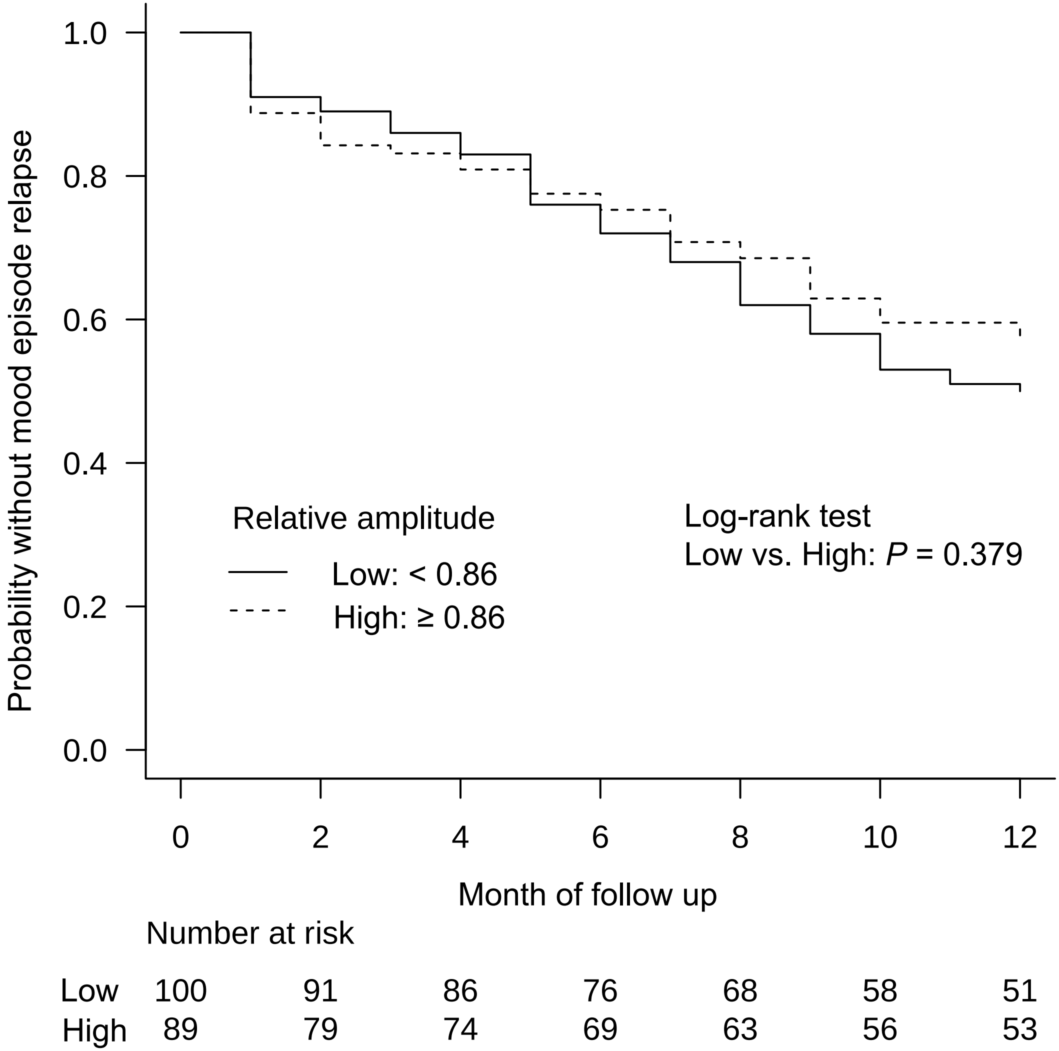
Probability without mood episode relapse
1.0
0.8
0.6
0.4
0.2
0.0
Relative amplitude
Low: < 0.86
High: ≥ 0.86
Log-rank test
Low vs. High: P = 0.379
0
2
4
6
8
10
12
Month of follow up
Number at risk
Low 100 91 86 76 68 58 51
High 89 79 74 69 63 56 53
