# Supplementary material for: Association between circadian activity rhythms and mood episode relapse in bipolar disorder: a 12-month prospective cohort study
Source: Transl Psychiatry. 2021 Oct 13;11:525. doi: 10.1038/s41398-021-01652-9 (PMC8514471; doi:10.1038/s41398-021-01652-9)
Supplement: Supplementary file 2 — Supplemental table 1 [file 41398_2021_1652_MOESM2_ESM.docx]

| **Supplemental table 1.** Cox proportional hazards analysis for mood episode relapse associated with circadian activity rhythm parameters | | | | | | | | |
| --- | --- | --- | --- | --- | --- | --- | --- | --- |
|  | Crude model | |  | Adjusted model 1 | |  | Adjusted model 2 | |
| Circadian activity rhythm parameters | HR (95% CI) | *P* |  | HR (95% CI) | *P* |  | HR (95% CI) | *P* |
| Cosinor analysis |  |  |  |  |  |  |  |  |
| MESOR, per counts/min | 0.994 (0.989–0.998) | 0.004 |  | 0.993 (0.988–0.997) | 0.003 |  | 0.993 (0.988–0.997) | 0.002 |
| Amplitude, per counts/min | 0.993 (0.988–0.998) | 0.005 |  | 0.992 (0.987–0.998) | 0.005 |  | 0.994 (0.988–0.999) | 0.020 |
| Acrophase, per hour | 1.143 (1.040–1.255) | 0.005 |  | 1.093 (0.985–1.213) | 0.095 |  | 1.066 (0.954–1.190) | 0.258 |
| The circadian activity rhythm periods of all participants were defined as 24-hours. Model 1 was adjusted for age and gender. Model 2 was adjusted for age, gender, residual mood symptoms, multiple mood episodes within 1 year before baseline assessment, total sleep time, sleep efficiency, and daytime illuminance. HR, hazard ratio; CI, confidence interval; MESOR, midline-estimating statistic of rhythm. | | | | | | | | |
