# Supplementary material for: Association between circadian activity rhythms and mood episode relapse in bipolar disorder: a 12-month prospective cohort study
Source: Transl Psychiatry. 2021 Oct 13;11:525. doi: 10.1038/s41398-021-01652-9 (PMC8514471; doi:10.1038/s41398-021-01652-9)
Supplement: Supplementary file 3 — Supplemental table 2 [file 41398_2021_1652_MOESM3_ESM.docx]

| **Supplemental table 2.** Cox proportional hazards analysis for depressive episode relapse associated with circadian activity rhythm parameters | | | | | | | | |
| --- | --- | --- | --- | --- | --- | --- | --- | --- |
|  | Crude model | |  | Adjusted model 1 | |  | Adjusted model 2 | |
| Circadian activity rhythm parameters | HR (95% CI) | *P* |  | HR (95% CI) | *P* |  | HR (95% CI) | *P* |
| Cosinor analysis |  |  |  |  |  |  |  |  |
| MESOR, per counts/min | 0.992 (0.987–0.997) | 0.001 |  | 0.991 (0.986–0.996) | 0.001 |  | 0.990 (0.985–0.996) | <0.001 |
| Amplitude, per counts/min | 0.992 (0.986–0.998) | 0.006 |  | 0.992 (0.986–0.998) | 0.007 |  | 0.993 (0.986–0.999) | 0.017 |
| Acrophase, per hour | 1.147 (1.034–1.272) | 0.009 |  | 1.110 (0.989–1.245) | 0.076 |  | 1.103 (0.976–1.247) | 0.115 |
| The circadian activity rhythm periods of all participants were defined as 24-hours. Model 1 was adjusted for age and gender. Model 2 was adjusted for age, gender, residual mood symptoms, multiple mood episodes within 1 year before baseline assessment, total sleep time, sleep efficiency, and daytime illuminance. HR, hazard ratio; CI, confidence interval; MESOR, midline-estimating statistic of rhythm. | | | | | | | | |
